# The Specialist in Medicines Development (SMD) as a Vocational Program in Pharmaceutical Medicine: The Japanese and Italian Experience

**DOI:** 10.3389/fphar.2020.00062

**Published:** 2020-02-25

**Authors:** Domenico Criscuolo, Kyoko Imamura, Ingrid Klingmann

**Affiliations:** ^1^International Federation of Associations of Pharmaceutical Physicians and Pharmaceutical Medicine, Woerden, Netherlands; ^2^Social Cooperating Program of IT Healthcare, Graduate School of Pharmaceutical Sciences, The University of Tokyo, Tokyo, Japan; ^3^PharmaTrain Federation, Brussels, Belgium

**Keywords:** vocational training program, pharmaceutical medicine, PharmaTrain, specialist in medicines development, IFAPP, SIMeF

## Abstract

The growing complexity of the drug development process requires globally recognized professionals who have not only completed the cognitive path of competence, i.e. the specialized post graduate course in Pharmaceutical Medicine, but suggests that these individuals should join a vocational training program in order to consolidate the seven competencies which characterize a competent Pharmaceutical Professional. The Specialist in Medicines Development (SMD) program developed by the IMI project PharmaTrain and further supported by the IMI project IMI-TRAIN can be considered a prototype vocational program. In order to test the SMD value, it was implemented in two countries, Japan and Italy. The preliminary results, after three years of its implementation, are here summarized, and some initial recommendations are offered to all other countries which may consider to establish this program.

## Introduction

Professionals with medical and non-medical backgrounds work in the complex environment of medicines development in different functions and are trained on-the-job, leading to an array of competencies across multiple domains (Silva et al., 2013). Some of them may have attended one of the university-based post-graduate diploma or master courses in Pharmaceutical Medicine, offered since many years in several European countries, and in Japan, South Africa and few other countries. However, there is a growing consensus that a diploma or master title in Pharmaceutical Medicine is just a starting point: professionals involved in the drug development process should know in depth its entire process, and also be able to apply their knowledge reliably and creatively in their daily working environment in order to drive innovative R&D efficiently. Such vocational training is defined as a “training that emphasizes skills and knowledge required for a particular job function” (https://www.skillsportal.co.za/content/what-exactly-vocational-training).

In the UK, Switzerland and Ireland a vocational program for physicians has been implemented, leading to a national medical board certification in Pharmaceutical Medicine. But no globally recognized educational path nor structured qualification process is available for physicians in other countries, and for not medically qualified professionals working in medicines development (Imamura et al., 2019). Indeed, it was recently published (Redfearn, 2018) that “certified principal investigators (CPIs) and clinical research coordinators (CRCs) do better work compared with their peers who hold no certification”: however this certification should be extended to all professionals involved in the drug development process, and should be harmonized.

One of the first projects established by the European Public-Private Partnership Innovative Medicines Initiative (www.imi.europa.eu) was the “PharmaTrain” project (2009–2014, Grant Agreement number 115013) (PharmaTrain, 2019). Its first objective was the syllabus harmonization and the quality improvement of postgraduate education in Pharmaceutical Medicine/Medicines Development Sciences, resulting in a PharmaTrain recognition system for diploma and master courses. The second objective was the development of a competency-based, workplace-centered education and training certification program in Medicines Development, comprising a knowledge base covering the PharmaTrain Syllabus for Medicines Development Science, delivered and assessed through modular curricula, and the acquisition and demonstration of competencies for medicines development across seven domains of the competency curriculum (Silva et al., 2013). Participants in this mentored, vocational program should acquire knowledge and competencies within a framework of assessment, appraisal and annual review of progress and achievements. On completion, participants will receive the SMD Certificate from the global PharmaTrain Certification Board (gPCB).

A pilot implementation of the SMD concept was funded by IMI from 2014 to 2016 as part of the IMI-EMTRAIN ENSO project “IMI-TRAIN” (Grant Agreement number 115015) (IMI-TRAIN, 2019). This project integrated the results and organizations of all training-related IMI projects with the aim to create an IMI Education and Training Infrastructure with a common IT portal to a joint platform for Continuous Professional Development (CPD) tools including new online training modules, and the SMD certification program as a role model for other specialist title certification programs like in safety sciences or pharmacovigilance and epidemiology. In a dedicated Work Package, the infrastructure for the SMD program was developed in cooperation between academic teaching institutions and pharmaceutical companies to enable national implementation by a local Pharmaceutical Medicine organization. Ideal candidates for the SMD program were either professionals with a significant experience (more than 4 years of professional activity in drug development) or professionals who, after obtaining the title of master/diploma in Pharmaceutical Medicine, were in the position to start their professional career in drug development.

The country selected for the SMD pilot implementation was Italy, due to its well established post-graduate education infrastructure in Pharmaceutical Medicine, a relevant pharmaceutical industry, the interest of the Italian Association of Pharmaceutical Medicine (SIMeF) to create such career development opportunities for their members, and the availability of two master courses (Catholic University of Rome and Bicocca University in Milan) which received the recognition of PharmaTrain Centres of Excellence (Criscuolo, 2010).

One of PharmaTrain's consortium partners was IFAPP, the International Federation of Associations of Pharmaceutical Physicians and Pharmaceutical Medicine, and another course recognized as a PharmaTrain Centre of Excellence was the master course in Pharmaceutical Medicine at the University of Osaka. The Japanese Association of Pharmaceutical Medicine (JAPhMed) expressed interest in joining the pilot initiative with their own resources.

Experiences made with implementation of the SMD concept in Italy and Japan are here illustrated and commented.

### The SMD Pilot Program in Japan

In accordance with global PharmaTrain SOPs, a national PharmaTrain Certification Board (nPCB) was established with representatives from JAPhMed, ACRP (Association of Clinical Research Professionals Japan), and EFPIA (European Federation of Pharmaceutical Industry Association Japan), in 2016. Upon initiation of the second year (2017), professors and directors from national universities and national medical centers joined the nPCB, which currently consists of eight members. The nPCB is established and acknowledged by the gPCB to meet the requirements of the local SMD program, according to legal or regulatory requirements, geography and culture. All nPCB members are qualified directors or have a master level, or above, in a life science or medical field, are actively working in or consulting in the area of medicines development science or pharmaceutical medicine, are experienced in at least two speciality domains of the SMD curriculum over a period of not less than 10 years, and are actively undertaking Continuing Professional Development (CPD) in their own area of competence.

Under the nPCB, a SEG (SMD Executive Group) was established by the nPCB, to undertake the executive actions of the nPCB to implement, monitor and administrate the SMD program. At present, eight members of the SEG regularly meet and discuss progress of the program and any issue to be discussed at the nPCB meetings, which are held on quarterly basis. In order to align with global directions and to standardize the methods of assessment, a series of SOPs were developed. Forms to report learning activities and their assessment results were also developed for standardized reporting. Since the development of the nPCB and the SEG, the program has been promoted on the JAPhMed website, showing program entry criteria, methods for regular review and assessment of competency by the mentor in the workplace, and the final certification to be granted by the gPCB.

To introduce this new program, ten participants were invited for a free submission, six of them from the industry and the other four from academia. Four new trainees and mentors are registered in the second year as fee-paying participants. The SMD program entry criteria require participants to have completed a formal education (BSc, MSc, MD, PharmD, RN, DV, PhD, or equivalent) in a discipline in life science or healthcare e.g. medical doctors, pharmacists, biologists, chemists, biometricians, certified nurses, as well as to hold a job related to research, development or medical marketing of medicinal products. At present, one trainee was assessed as competent by her mentor and her records were examined by the SEG with a third party review by experts. Having reviewed her assessment records, the nPCB recommended to the gPCB to assign the SMD title. After review by gPCB, this trainee was certified as the first SMD since the development of this international program.

The program asks trainees to complete theoretical training (specialty knowledge base) in medicines development in an accredited course covering the entire PharmaTrain Syllabus (The PharmaTrain Federation), with assessments and certified outcome, as well as the evidence over 4-year period of gaining practical training and competencies in medicines development. This has been the main difficulty for the SMD program in Japan, because there was no systematic education in medicines development until 2013 when the first PharmaTrain educational course was developed in collaboration with the Osaka University. Although there are many relevant courses and lectures in this area, most of them lack assessments and do not fully cover the PharmaTrain Syllabus. Therefore, after the baseline assessment of competency, the trainee and the mentor had to develop a plan to fill the gap in education. The second difficulty that most trainees have experienced are the career changes of mentors and/or themselves. Once it happens, trainees must find alternative mentors who can succeed to supervise their program, but it is not always easy to identify dedicated mentors in their workplaces. Although the Japanese job market is less fluid as compared to other countries, increasing numbers of corporate mergers and acquisitions, as well as organizational changes are the constant risk for the SMD program.

### The SMD Pilot Program in Italy

In Italy, Regulatory Authorities have addressed, at least in part, the need for structured post-graduate education in Pharmaceutical Medicine for professionals working in this environment. In fact, according to an Italian law dated 2008 (Italian Ministry of Health, 2008), all professionals working in a CRO must follow a dedicated training program, which can be considered as completed by the achievement of a University master title in Pharmaceutical Medicine. This law was instrumental for the implementation of several master courses in Pharmaceutical Medicine in several Universities all over the country (Criscuolo, 2017). Currently, the number of professionals in Pharmaceutical Medicine with a master title is approaching 1500. These professionals are the ideal candidates for the SMD program: in fact, the gPCB decided that Italian professionals holding a master title, which means that they achieved a significant background knowledge in drug research and development over a period of 1.5 years and performed a work-place based stage of at least 6 months, need only to be followed up in a mentored fashion during 2 years of their initial professional activity to achieve the SMD title.

The initial process of the SMD program in Italy was very similar to the steps established by our Japanese colleagues: the main difference is in the nPCB composition. In fact, in order to get more visibility and an independent body, SIMeF opened this Board to several organizations with whom there is a long lasting collaboration. SIMeF identified, among its executives, four senior members for the nPCB, who had the task to support all planned activities. But the nPCB was also composed by the following additional members: one delegate from AIFA (the Italian Drug Agency), one delegate from each of the two Universities where there is a master in Pharmaceutical Medicine which got the PharmaTrain recognition of Centre of Excellence (Rome Catholic University and Milano Bicocca University), one representative of SIF (the Italian Association of Pharmacologists), one representative of FADOI (the Italian Association of Internal Medicine) and one representative of a patients association. In total, the Italian nPCB is therefore composed of ten members, and the majority of this Board is made of independent professionals.

The first meeting of the nPCB was held in the premises of AIFA, the Italian Drug Agency, in order to give more value to the event, and to underline the independent role of this body: further meetings were and are held every six months, and are mainly arranged *via* teleconference. Great efforts were put in place to disseminate the information about the SMD program, and to attract registrations: a very low fee was also enforced (first year free, second and further years at 500 euro), having in mind that most applicants were supposed to pay this fee by themselves. All relevant information about the SMD opportunity was posted on the SIMeF website on a dedicated page; in addition, at every SIMeF seminar, an SMD leaflet was distributed to all participants; finally, in every issue of the SIMeF bimonthly newsletter, an advertisement about the SMD opportunity is published.

Notwithstanding these significant efforts, and also considering the large number of potential applicants holding a master title, two years after the implementation of the SMD program in Italy, we have only four professionals who registered. Three of them are from the same pharmaceutical company, working in the Medical Affairs dept, and their registrations were strongly stimulated by their Medical Director, who greatly appreciated the program's aims and contents. The fourth applicant is a professional holding the master title, who joined the program in order to get a more pragmatic training in Pharmaceutical Medicine.

## Discussion

The preparation of the SMD concept started in PharmaTrain in 2013 but was only completed in the IMITRAIN project in 2016, therefore the two pilot experiences of the SMD implementation, in Italy and in Japan, started only 3 years ago. Therefore, it may be too early to draw some conclusions. However, some initial comments may be appropriate, having also in mind that additional National Associations of Pharmaceutical Medicine may be interested to support this program in their countries.

Indeed, the basic idea of the vocational SMD program is very appealing: professionals educated theoretically in Pharmaceutical Medicine may gain great competence from the SMD program, because they receive the unique opportunity, while on their job, to gain experience in practice about the full process of drug development, to become familiar with the consequences of decisions made during this path and to have to interact with professionals of several areas, in order to complete their understanding in the seven domains required for being a competent pharmaceutical professional (Silva et al., 2013; Dubois et al., 2016; Criscuolo, 2017; Imamura et al., 2019) (see **Table 1**).

**Table 1:** Statement of competences in pharmaceutical medicine. IFAPP/PharmaTrain 2012 [from Silva et al. (2013)].

**Domain I:** Discovery medicine and early development. To identify unmet therapeutic needs, evaluate the evidence for a new candidate for clinical development and design a Clinical Development Plan for a Target Product Profile.
**Domain II:** Clinical development and clinical trials. To design, execute and evaluate exploratory and confirmatory clinical trials and prepare manuscripts or reports for publication and regulatory submissions.
**Domain III:** Medicines regulation. To interpret effectively the regulatory requirements for the clinical development of a new drug through the product life-cycle to ensure its appropriate therapeutic use and proper risk management.
**Domain IV:** Drug safety surveillance. To evaluate the choice, application and analysis of post-authorization surveillance methods to meet the requirements of national/international agencies for proper information and risk minimization to patients and clinical trial subjects.
**Domain V:** Ethics and subject protection. To combine the principles of clinical research and business ethics for the conduct of clinical trials and commercial operations within the organization.
**Domain VI:** Healthcare marketplace. To appraise the pharmaceutical business activities in the healthcare environment to ensure that they remain appropriate, ethical and legal to keep the welfare of patients and subjects at the forefront of decision-making in the promotion of medicines and design of clinical trials.
**Domain VII:** Communications and management. To interpret the principles and practices of people management and leadership, using effective communication techniques and interpersonal skills to influence key stakeholders and achieve the scientific and business objectives.

The recognition of the benefits of a candidate's SMD certification to an employer takes time; and also the relevance for attracting high potential professionals by offering the SMD mentoring program in their organization still needs to be fully appreciated. Therefore, National Associations of Pharmaceutical Medicine, universities and industry associations which are promoting this initiative must continue to stimulate professionals, especially the younger ones who have obtained a master title in Pharmaceutical Medicine, to join the SMD program, and to make employers aware of the relevance of these professionals for their organization's success. In the long run this will clearly demonstrate the significant importance of having competent professionals devoted to drug development, in a worldwide environment.

Finally, the SMD program is an important additional step to achieve the PharmaTrain vision of modern competence which says “Better trained postgraduate professionals working in medicines development and regulation worldwide produce better medicines”.

## Author Contributions

DC conceived the manuscript, and wrote *Introduction*, the section about Italy implementation, and conclusions. KI contributed to the idea of the manuscript, and wrote the section about Japan. IK contributed to the idea of the manuscript, and wrote the section about PharmaTrain and IMI roles in SMD preparation.

## Funding

The SMD project was in part funded by grant 115013 from IMI and by grant 115015 from IMI-TRAIN.

## Conflict of Interest

The authors declare that the research was conducted in the absence of any commercial or financial relationships that could be construed as a potential conflict of interest.
